# The Emotional Landscape of Pregnancy and Postpartum during the COVID-19 Pandemic in Italy: A Mixed-Method Analysis Using Artificial Intelligence

**DOI:** 10.3390/jcm12196140

**Published:** 2023-09-23

**Authors:** Claudia Ravaldi, Laura Mosconi, Roberto Bonaiuti, Alfredo Vannacci

**Affiliations:** PeaRL—Perinatal Research Laboratory, CiaoLapo Foundation, Department of Neurosciences, Psychology, Drug Research and Child Health, University of Florence, 50121 Florence, Italy; claudia.ravaldi@unifi.it (C.R.); laura.mosconi@unifi.it (L.M.); roberto.bonaiuti@unifi.it (R.B.)

**Keywords:** COVID-19 and pregnancy, management, prevention

## Abstract

The COVID-19 pandemic affected the perinatal emotional landscape in Italy, a country that had high mortality and implemented a strict lockdown during the pandemic. This study explores the emotions and challenges of pregnant and postpartum women during the pandemic, using AI-based mixed methods. The study analyzed 1774 women from the national survey COVID-ASSESS: 1136 pregnant and 638 postpartum women. The survey had qualitative questions on emotions and feelings related to birth, communication with healthcare professionals, media, and peers and family. We used natural language processing and machine learning to classify emotions, identify themes, and extract citations from the data. Fear and anxiety replaced joy as dominant emotions during the pandemic: trust and joy decreased by 49.3% and 36.4%, respectively, while sadness and fear increased by 52.3% and 49.3%, respectively. The pandemic also induced loneliness, isolation, frustration, and anger. Women faced challenges related to birth, communication with HCPs, media, and peers and family. They also used coping strategies such as self-care, news limitation, and trying to cultivate gratitude and hope. This study provides a comprehensive exploration of the perinatal emotional landscape of Italian women during the pandemic. The findings underscore the significant psychological impact of the pandemic and also highlight women’s resilience and coping strategies.

## 1. Introduction

In the early months of the COVID-19 pandemic, Italy was uniquely positioned as the first Western nation to be severely impacted by the virus. This period was characterized by a high degree of uncertainty and fear, as little was known about the infection and its potential effects. The rapid spread of the epidemic and the ensuing lockdown measures had a profound impact on the mental health of the population, leading to widespread psychological distress and increased mental health risks [1,2].

The societal burden of this situation was substantial. The healthcare system, already strained by the direct effects of the virus [1], faced additional pressure from the increased demand for mental health services [3]. Moreover, the economic consequences of the pandemic, including job loss and financial instability, further exacerbated mental health issues and contributed to a cycle of stress and anxiety [4].

The disruption of healthcare services afforded by the pandemic also included prenatal and postnatal care [5]. In this context, more vulnerable populations, such as pregnant and postpartum women, were particularly affected. Pregnant women with COVID-19 infection had an increased mortality rate and other adverse outcomes such as higher rates of preterm birth and pre-eclampsia [6]. Furthermore, the uncertainty and fear associated with COVID-19, combined with the isolation and social distancing measures implemented to control the spread of the virus, created additional stressors that increased the risk of developing depression and anxiety symptoms during pregnancy [7,8,9]. In fact, the perinatal period, by itself, is characterized by significant physiological and psychological changes and challenges, which can place women at higher risk for mental health difficulties, including depression, anxiety, and post-traumatic stress disorder (PTSD) [10,11,12]. Moreover, the subjective negative impact of COVID-19 has been associated with more dysfunctional coping and less emotion-focused coping [13]. The impact of COVID-19 on perinatal mental health highlights the need for additional support and resources [9,14].

Our previous research, conducted during the initial phase of the COVID-19 lockdown in Italy, provided preliminary insights into the emotional experiences of pregnant and postpartum women [15,16,17]. These studies found that pregnant women experienced significant psychological distress during the pandemic, including symptoms of depression, anxiety, and stress. Furthermore, exclusive breastfeeding was found to serve as an independent protective factor for women’s mental health during the pandemic [17].

However, a more comprehensive analysis of the ongoing nature of the pandemic and its evolving impact is needed. The present study aims to delve deeper into the emotional landscape of pregnant and postpartum women during the first months of the pandemic, focusing on the prevalence of various emotions and the coping strategies employed. Here, we aim to build upon our previous findings and provide a more nuanced understanding of the emotional experiences of pregnant and postpartum women during this global crisis. 

Understanding these emotional responses and coping mechanisms is crucial for developing effective interventions to support perinatal mental health during such crises. The present study aims to contribute to this understanding by examining, through a qualitative analysis, the emotional experiences and coping strategies of pregnant and postpartum women in Italy during the COVID-19 pandemic.

## 2. Materials and Methods

### 2.1. Survey

COVID-ASSESS is a cross-sectional study based on a survey administered during the first wave of the COVID-19 pandemic in Italy. The survey was distributed via CiaoLapo, an Italian charity for perinatal health support, using existing networks and support groups across Italy. Participants self-selected to complete the survey and participation was voluntary. The survey was launched in March 2020, and data were collected until May 2020. Each participant gave their explicit consent in an online form before enrolment. The survey was uploaded as an online tool using the Surveymonkey platform (www.surveymonkey.com) (accessed on 20 September 2023) and comprised the following sections: (A) socio-demographic information, (B) previous losses, personal and family history of psychological disorders, (C) birth expectations before and after COVID-19, (D) concerns regarding pandemic consequences, (E) postpartum health and infant feeding, (F) perception of media and health professionals’ information and communication on COVID-19, and (G) psychometric evaluation tests. Methodological details, full text from the survey, and raw data have already been published [18].

This paper presents a secondary analysis of the national survey COVID-ASSESS that originally included 2448 women, of whom 1307 were pregnant and 1141 postpartum. Participants were considered eligible to be included in this post hoc analysis if they answered at least one of the qualitative questions of the survey. Full text of the survey, a general description of results, and complete raw data have already been published [18] and are freely available in an online repository [19].

### 2.2. Classification of Emotions

We employed the GPT-3.5-turbo (Generative Pre-trained Transformer) model developed by OpenAI to classify emotions in text without fine-tuning the model specifically for this task. GPT-3.5 is a state-of-the-art natural language processing model pre-trained on a large corpus of text data, primarily designed for natural language understanding and generation tasks. However, large-scale pre-training allows it to capture a wide range of linguistic features that can be useful for emotion classification [20,21].

To adapt GPT-3.5 for emotion classification without fine-tuning, we used a zero-shot learning approach. This method involves formulating the input prompt in a way that guides the model to provide the desired output, in our case, the emotion(s) associated with the text. The following steps were followed for emotion classification using GPT-3.5:

1. **Preprocessing**: any given input text was preprocessed by removing irrelevant information, text was converted to lowercase, words were tokenized, and stop words and punctuation were removed.
2. **Prompt formulation**: a prompt was formulated to include the preprocessed input text and the model was asked to identify the emotion(s) associated with it. The prompt explicitly listed the eight target emotions (anger, anticipation, joy, trust, fear, surprise, sadness, and disgust) and the model was instructed to select the most appropriate one(s). In particular, we used the Google Sheet plugin “GPT for Sheets” by Talarian (https://gptforwork.com/) (accessed on 20 September 2023), with the following prompt: “Perform sentiment analysis and input 1 or 0 separated by commas for each of the following emotions: anger, anticipation, joy, trust, fear, surprise, sadness and disgust. Example: if all are present 1, 1, 1, 1, 1, 1, 1, 1; if all are absent 0, 0, 0, 0, 0, 0, 0, 0; if only anger is present 1, 0, 0, 0, 0, 0, 0, 0, etc…”.
3. **Inference**: GPT-3.5-turbo generated an output based on its pre-trained knowledge and the context provided by the prompt for each one of the 1774 rows. The output was stored as a string of comma-separated binary values in two Google sheet cells, one for ‘pre-COVID emotions’ and one for ‘post-COVID emotions’.
4. **Post-processing**: the Google sheet document was then imported into StataBE 18 (StataCorp) and the emotion strings were converted into regular fields for each emotion coded as absent (0) or present (1), both before and after the pandemic.

We also experimented with the more recent GPT-4 model for emotion classification [22]. However, we encountered several challenges with GPT-4. First, it was more costly to run than GPT-3.5-turbo. For example, it required USD 0.03 per 1 K token, while GPT-3.5-turbo only required USD 0.002 per 1 K token. Second, it was much slower than GPT-3.5-turbo. For instance, it took an average of 30 s to process one row of data, while GPT-3.5-turbo took only 3 s. To evaluate the potential benefits of using GPT-4, we performed a sensitivity analysis on a random sample of 20 rows from our dataset. This analysis showed no significant difference in the emotion classification accuracy between GPT-3.5-turbo and GPT-4, with a concordance of 99.4%. Therefore, we decided to use GPT-3.5-turbo for our study due to its lower cost, faster speed, and comparable performance.

### 2.3. Classification of Themes

In order to identify and analyze the main themes present within the given text, we employed the GPT-4 language model, with a natural language processing approach. Analyses were conducted providing the text of the survey in the platform https://chat.openai.com/ (accessed on 20 September 2023) using GPT-4 as a model. Before input, all sets of answers were divided into blocks of about 1000 words, and the following steps were taken to perform the analysis using the AI model:

1. **Tokenization**. Tokenization is the process of segmenting the input text into individual units called tokens, which can be words, phrases, or punctuation marks. This is an essential pre-processing step in NLP. The GPT model tokenized the text to facilitate further analysis, as it allowed for easier identification and quantification of the most recurring words and phrases. This was achieved using the model’s internal tokenization algorithms, which have been trained to recognize and separate the constituent elements of different languages, including Italian.
2. **Frequency Analysis**. After tokenization, the GPT model conducted a frequency analysis of the words and phrases in the text. This involved counting the occurrences of each token and ranking them based on their frequency. This step was crucial for determining the prominence and significance of particular words and phrases in the text, which helped in identifying the main themes. The frequency analysis was performed by leveraging the model’s internal algorithms for counting and ranking tokens.
3. **Semantic Analysis**. In order to group related words and phrases into overarching themes, the GPT model performed semantic analysis. This involved leveraging its pretrained knowledge of language and understanding of semantics to identify words and phrases with similar meanings or connotations. The model used its internal algorithms to analyze the relationships between tokens, taking into account their context and meaning, and grouped them into themes that represented the broader concepts present in the text.
4. **Scoring**. The GPT model assigned a score to each theme based on the frequency of the words and phrases within the theme. Once the text was analyzed to identify words and phrases that were indicative of the themes in question, words were counted, and the proportion of words associated with each theme was calculated as a percentage of the total number of words in the text. This percentage was then mapped to a scale of 0 to 10, where a score of 0 represented no presence of the theme in the text, and a score of 10 represented extremely high prominence of the theme. This scoring system allowed for a relative comparison of the prominence of different themes within the text, based on the frequency and distribution of indicative words and phrases.
5. **Textual Citations**. To provide evidence for each identified theme, the GPT model extracted relevant textual citations in Italian from the original text. This process involved identifying sentences or phrases that exemplified the main ideas of each theme while ensuring that the citations were representative of the broader context in which the theme appeared. The model leveraged its understanding of language and context to select appropriate citations that effectively illustrated the themes and their significance within the text. Citations were manually checked for accuracy and translated into English by the authors.

### 2.4. Thematic Map

A thematic map was created with the software Xmind (v22.11 Xmind LTD) based on four factors: the frequency of themes, the connections between themes identified by GPT-4 and explained in the Section 4, the connections underlined by literature, and the researchers’ experience in qualitative data analysis. 

## 3. Results

### 3.1. Characteristics of the Sample

The study sample consisted of 1774 women, of whom 1136 (64.0%) were pregnant and 638 (36.0%) were postpartum women. The mean age of the sample was 33.6 years (SD = 4.8), and the majority of the women had a high level of education (45.3% had a first stage of tertiary education and 16.2% had a second stage of tertiary education). The pregnant women were distributed across the three trimesters of pregnancy, with 47.9% in the third trimester, 42.2% in the second trimester, and 9.9% in the first trimester. The postpartum women had babies with a mean age of 2.7 months (SD = 1.8), ranging from less than 1.4 months to more than 4 months. Most of the women were multigravidae (64.2%), and 38.0% had a history of previous pregnancy loss. Nearly half of the women (44.5%) reported having a psychopathological history, and the majority of them (51.1%) had no other children. No significant difference in baseline characteristics was present between pregnant and postpartum women. More detailed information is available in Table 1.

### 3.2. Emotions Related to Birth

**Figure 1:**
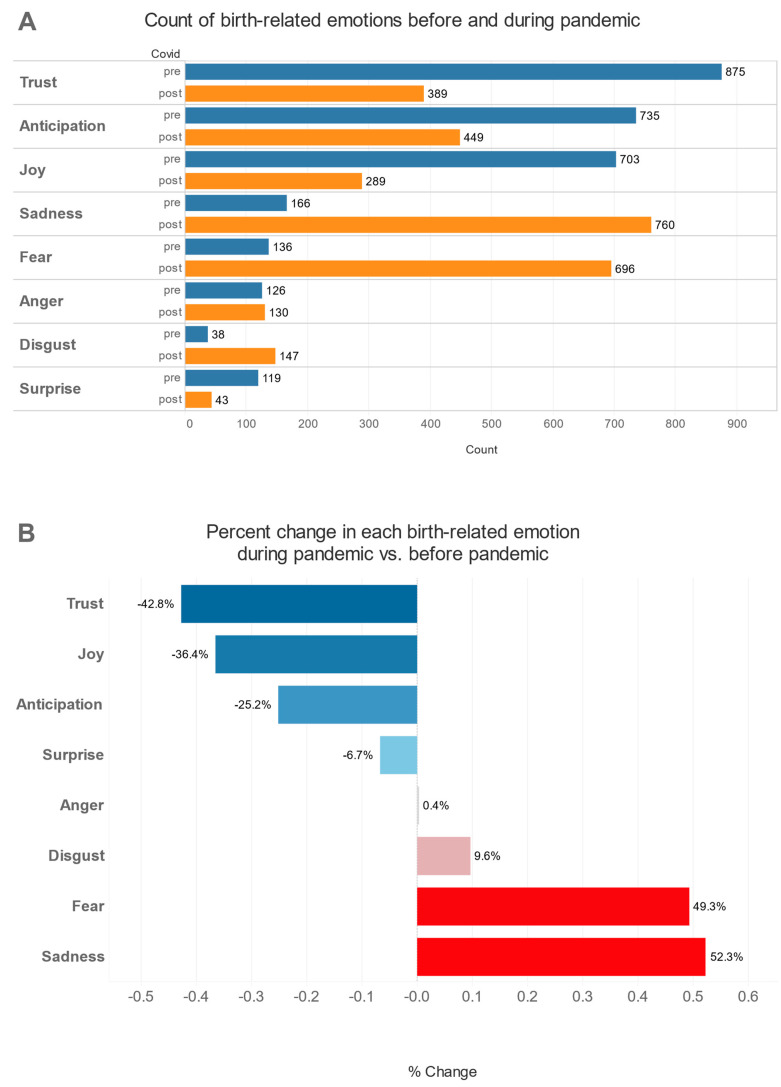
Changes in emotions before and during the pandemic. (**A**) shows women’s emotions before and during the pandemic. (**B**) shows how emotions changed in percentage during the pandemic as compared to before it.

Figure 1 shows that the pandemic had a significant impact on the birth-related emotions of pregnant women. Before the pandemic, the most common emotions were trust (875 women), anticipation (735 women), and joy (703 women). During the pandemic, the most common emotions were sadness (760 people), fear (696 people), and anticipation (449 women) (Figure 1A). In particular, the emotions that decreased the most were trust (−49.3%) and joy (−36.4%), while the emotions that increased the most were sadness (+52.3%) and fear (+49.3%) (Figure 1B).

**Figure 2:**
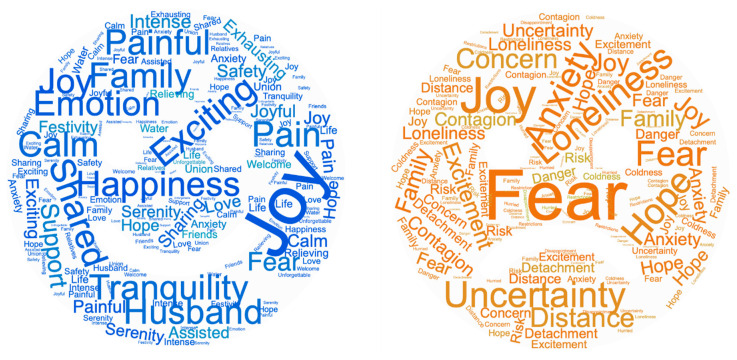
Word clouds of women’s emotions before and during the pandemic.

The main concepts expressed by women regarding birth before and during the pandemic are graphically depicted in Figure 2 as word clouds. The most frequent words and relative weights used to create the word clouds are reported in Supplementary Table S1.

### 3.3. Communication

The blocks of texts analyzed consisted of (a) 5434 words from phrases describing women’s experiences with healthcare professionals’ communication, (b) 3828 words from phrases describing women’s experiences with media communication, and (c) 3767 words from phrases describing women’s experiences with peers (family, friends, colleagues, etc.) during the acute phase of the pandemic. We also included a big block of 46,542 words, derived from the open-ended questions in which responders could write any reflection they wanted, without specific guidance.

A total of 59,571 words, corresponding to 341,964 characters, were analyzed. Based on the repetition of words and concepts, the themes were automatically graded by the AI on a scale from 0 to 10, with higher scores indicating greater recurrence within the text. Quotes have been provided as examples of words and phrases suggesting each theme.

**Figure 3:**
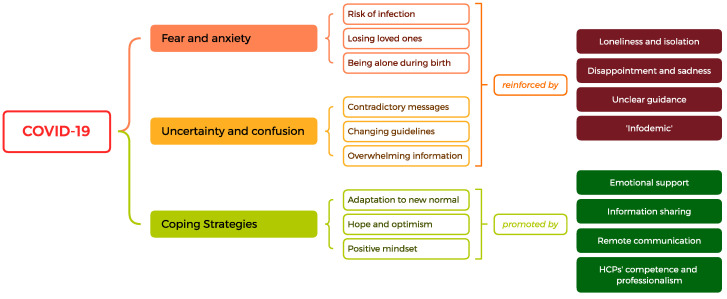
Thematic map of the main emerging themes.

A thematic map showing the synthesis of the relationships between the main themes found is shown in Figure 3.

#### 3.3.1. HCPs and Communication

Five themes have emerged as prominent aspects of the communication between healthcare professionals and the public during the acute phase of the pandemic:

1. **Fear and Anxiety** (Score 8/10). Fear and anxiety were the most common emotions expressed throughout the text. Women reported feeling scared, uncertain, and panicked due to the pandemic and the information provided by healthcare professionals. This theme was prevalent across all the text. Examples of words used include the following: “fear”, “anxiety”, “panic”, “worry”, “apprehension”, “tension”, “dread”, “terror”, “anguish”, and “insecurity”.
2. **Uncertainty and Confusion** (Score 7/10). Many respondents described the communication from healthcare professionals as unclear, contradictory, or insufficient, leading to feelings of confusion and uncertainty. This theme was consistently present in the text. Examples of words used include the following: “uncertainty”, “confusion”, “disorientation”, “doubt”, “insecurity”, “vagueness”, “ambiguity”, “disinformation”, “hesitation”, and “contradictions”.
3. **Emotional Support and Reassurance** (Score 6/10). Some respondents mentioned the importance of emotional support and reassurance provided by healthcare professionals. Women reported that clear, direct, comforting and empathetic communication from their HCPs helped foster a sense of hope, even in the face of uncertainty and fear. Seeking support from HCPs was used as a coping strategy that enabled women to focus on the positive aspects of their care. Examples of terms used include the following: “emotional support”, “reassurance”, “comfort”, “calmness”, “serenity”, “hope”, “encouragement”, “understanding”, “empathy”, and “positivity”.
4. **Professionalism and Competence** (Score 5/10). Several individuals highlighted the professionalism and competence of healthcare workers during the pandemic. This theme recurred across the text, emphasizing the importance of skilled professionals in handling the crisis. Examples of words used include the following: “professionalism”, “competence”, “expertise”, “prepared”, “dedication”, “commitment”, “excellence”, “skilled”, “efficient”, “focused”, and “humanity”.
5. **Distance and Detachment** (Score 4/10). The text also reflected a sense of distance and detachment, both in terms of the physical limitations imposed by the pandemic and the emotional disconnection experienced by some women in their interactions with healthcare professionals. Examples of terms used include the following: “distance”, “detachment”, “isolation”, “separation”, “limited contact”, “disconnection”, “remote”, and “no feeling”.

#### 3.3.2. Media and Communication

Four main themes were identified describing the communication by the media during the acute phase of the pandemic:

1. **Fear and Anxiety** (Score: 10/10). The most prominent theme in the text is the sense of panic and anxiety induced by media communication during the pandemic. Respondents frequently mentioned feeling overwhelmed by the constant bombardment of alarming and sensationalist news. They reported feelings of fear, unease, and insecurity, which negatively impacted their mental health and well-being. Examples of words used include the following: “panic”, “anxiety”, “terror”, “fear”, “insecurity”, “overwhelmed”, “alarming”, and “sensationalist news”.
2. **Confusion and Contradictions** (Score: 8/10). Another significant theme is the confusion and contradictions in the media’s messaging. Participants expressed frustration with the lack of clarity and consistency in the information provided, which led to uncertainty and doubt about the situation. Examples of terms used include the following: “confusion”, “contradictions”, “inconsistencies”, “mixed messages”, “unclear guidance”, “ambiguity”, “discrepancies”, “misinformation”, “divergent opinions”, and “lack of clarity”.
3. **Sensationalism and Alarmism** (Score: 7/10). Many respondents criticized the media’s sensationalist and alarmist approach, which exacerbated feelings of panic and anxiety. They accused the media of exaggerating the situation, focusing on negative aspects, and employing a fear-based narrative to capture attention. Many women perceive a disproportionate emphasis on extreme cases, dire projections, and tragic stories while neglecting to provide context or balance with more positive or hopeful information. Such reporting practices were perceived as profit-driven, prioritizing viewer engagement and clickbait headlines over accurate and responsible journalism. As a coping strategy, women tried to limit exposure to distressing news, seeking out positive news stories. Examples of terms used include the following: “sensationalism”, “alarmism”, “exaggeration”, “hype”, “fearmongering”, “over-dramatization”, “catastrophizing”, “scaremongering”, “apocalyptic language”, and “inflated claims”.
4. **Misinformation and Inaccuracy** (Score: 6/10). The final theme we identified is the prevalence of misinformation and inaccuracy in media reporting. Participants mentioned issues with fake news, untrustworthy sources, and imprecise information that contributed to a chaotic and disorienting information landscape. Examples of terms used include the following: “misinformation”, “inaccuracy”, “false claims”, “unverified reports”, “disinformation”, “rumors”, “distorted facts”, “misleading statements”, “unreliable sources”, and “fabricated stories”.

#### 3.3.3. Peers and Communication

Five main themes were identified describing repsondents’ communication with peers (family, relatives, friends, colleagues) during the acute phase of the pandemic:

1. **Remote Communication** (Score: 8.5/10). The most recurrent theme was the reliance on remote communication methods, including phone calls, video calls, and messaging apps, as face-to-face interactions were limited. Participants often mentioned using these technologies to maintain social connections and exchange support during the pandemic. Examples of terms used include the following: “video calls”, “phone calls”, “messaging apps”, “online platforms”, “virtual meetings”, “text messages”, “social media”, and “email”.
2. **Adaptation and Coping Strategies** (Score: 7.5/10). Adjusting to the new normal and coping strategies emerged as a main theme in participants’ descriptions of their experiences during the pandemic. Among the most cited strategies were seeking emotional support from family, friends, and colleagues, as well as engaging in activities to distract from stress and uncertainty. Virtual connections through video calls and social media proved invaluable for maintaining relationships and alleviating feelings of isolation. Additionally, adopting positive mindsets, focusing on self-care, and staying informed were recurring coping techniques. Examples of terms used include the following: “adaptation”, “modifying”, “coping”, “reinventing”, “emotional support”, “distraction”, “virtual connection”, “positivity”, and “helping others”.
3. **Emotional Support** (Score: 7/10). Participants frequently discussed the importance of providing and receiving emotional support during this challenging period. Sharing feelings, thoughts, and concerns with their peers was a way to alleviate stress and anxiety related to the pandemic. Examples of terms used include the following: “empathy”, “comfort”, “reassurance”, “kindness”, “encouragement”, “caring”, “understanding”, “compassion”, “love”, and “listening”.
4. **Information Sharing** (Score: 6/10). Another significant theme was the exchange of information about the pandemic, such as updates on restrictions, health guidelines, and the availability of essential resources. Participants often highlighted how sharing information with their peers helped them stay informed and cope with the rapidly changing situation. Examples of words used include the following: “updates”, “news”, “facts”, “details”, “guidelines”, “recommendations”, “protocols”, “procedures”, “instructions”, and “advice”.
5. **Adaptation to the New Normal** (Score: 5.5/10). Participants also mentioned adjusting to the new normal, as the pandemic forced them to modify their communication habits and routines. This theme covered the adoption of new technologies, the struggle to maintain relationships, and the development of new ways of socializing. Examples of words used include the following: “adjusting”, “adapting”, “modifying”, “flexibility”, “changing”, “coping”, “reinventing”, “reshaping”, “reimagining”, and “transforming”.
6. **Fear and Anxiety** (Score: 5.5./10). Although less prevalent than in the other sections, the theme of fear and anxiety is also present in the Peers section, particularly related to the risk of infection from social interactions. Women expressed concerns about their peers’ and family members’ adherence to safety guidelines, which might expose them and their children to the virus. Examples of words used include the following: “scared”, “worried”, “anxious”, “stressed”, “concerned”, and “nervous”.

#### 3.3.4. Open-Ended Questions

Four main themes were further identified from the open-ended questions:

1. **Fear and anxiety** (Score: 9/10). Again, this theme was the most prominent in the text, as many women express their fear and anxiety about various aspects of their situation, such as getting infected, losing their loved ones, being alone during labor, facing economic difficulties, and not knowing when the pandemic will end. Some examples of textual quotations include “I think about suicide rather than going to the hospital and risking contracting COVID”, “I’m afraid I won’t be able to protect my little one”, “I’m scared because my childbirth would take place in less than 2 months and here we are talking about a virus that we don’t know much about”, and “I’m afraid that life will never go back to how it was before”.
2. **Loneliness and isolation** (Score: 8/10). This theme is also very frequent in the text, as many women miss the lack of social contact and support from their family and friends, especially during a delicate moment like pregnancy or breastfeeding. Some examples of textual quotations include “I feel emotionally alone, I have no one, apart from my partner, with whom to share anxieties, fears, but also joys and progress”, “I miss my family and I’m sorry that they are losing the first months of my baby”, and “I miss being able to see my parents and take my son to the park”.
3. **Gratitude and hope** (Score: 6/10). This theme is less frequent but still present in the text, as some women express their gratitude and hope for their situation, such as having a healthy baby or a supportive partner, or express hope for the future. Some examples of textual quotations include “I feel lucky because, unlike other colleagues, I can stay at home and not risk it”, “I’m grateful to have a baby that makes us happy, me and my husband, and fills our little house with love”, and “I hope that at least some women will understand how sick the world is because of globalization/pollution, too much work and how important it is to have friends and family”.
4. **Disappointment and sadness** (Score: 5/10). This theme is also present in the text, as some women express their disappointment and sadness at not being able to live their pregnancy or breastfeeding as they had imagined or planned due to the restrictions and limitations imposed by the pandemic. Some examples of textual quotations include “I wish I could live this pregnancy differently....especially sharing it closely with all the women who are important to me”, “I’m sad because no relative has been able to meet my son”, “The fear takes away from me the enthusiasm of pregnancy”, and “I miss being able to go swimming and go out with my family”.

## 4. Discussion

### 4.1. Emotions Regarding Childbirth

In the context of emotions related to birth, the themes of trust, joy, sadness, and fear are particularly salient. These emotions are not isolated but are interconnected and influenced by various factors, including the communication and support received from healthcare professionals, media, and peers.

In particular, the findings of this study are in line with those of our preliminary research conducted in a sample of 200 pregnant women during the very first phase of COVID-19 lockdown in Italy in April 2020 [15]. In particular, with this further analysis, conducted on a sample more than five times larger, we can confirm that ‘joy’ was the most prevalent emotion expressed before COVID-19, while fear was the most prevalent during the acute phase of the COVID-19 pandemic. During the pandemic, the joy associated with the anticipation of birth and motherhood was overshadowed by fear and uncertainty. This shift in emotional landscape underscores the profound impact of external factors, such as a global health crisis, on the emotional well-being of pregnant women. As mentioned before, this resonates with the increased risk of having a maternal and neonatal adverse outcome with a SARS-CoV-2 infection [23]. Furthermore, other findings of literature underlined that unexpected complications during childbirth, including those brought about by external circumstances, can lead to negative emotions and a sense of grief [24,25].

The negative effects of the pandemic on the emotions and expectations of pregnant women in Italy also lasted beyond the first lockdown phase. These findings are consistent with a study by Smorti et al. (2022), which found that the COVID-19 social restrictions increased the risk of depression in Italian women with low-risk pregnancy compared to the previous period [26]. This study also found that women with high-risk pregnancy used hospitalization “as a resource to find a social support network with other pregnant women and to be reassured on the clinical ongoing of pregnancy” [26]. 

### 4.2. Communication and Open-Ended Questions: The Main EMERGING Themes

Fear and anxiety were prominent emotions expressed by women. Women felt concerned about the potential risk of infection during hospital visits, about the safety of their unborn or newborn children (fear of losing loved ones), and about being alone during childbirth. Moreover, a study conducted by Azoulay et al. (2021) reported high levels of anxiety among healthcare providers, driven by fear of infection and the pressure of managing the COVID-19 surge [27]. This shared anxiety could potentially impact the communication between HCPs and patients, further exacerbating the patients’ emotions. When discussing the media, women expressed fear and anxiety stemming from the overwhelming amount of information, often contradictory and inaccurate, about the virus. This situation could be considered an infodemic characterized by uncertainty and confusion due to misinformation and inaccuracy as well as alarmism and sensationalism [28] during a disease outbreak. This is consistent with the literature, which suggests that the spread of information about COVID-19 through social media platforms has created fears that may not be entirely justified [29]. Moreover, infodemics lead to mistrust in HCPs and undermine the public health response. Regarding peers and family, fear and anxiety were also present, often related to the potential risk of infection from social interactions and to the possibility of being alone during labor and childbirth. In this sense, this theme shows a connection with the theme of loneliness and isolation.

Uncertainty and confusion were often present in the context of HCPs, tied to the rapidly evolving nature of the pandemic and the subsequent changes in healthcare protocols. This was further exacerbated by the fact that the scientific understanding of COVID-19 was a work in progress leading to repeated changes in guidelines and recommendations, often accompanied by contradictory messages and overwhelming information. This aligns with the paper of Koffman et al. (2020), who noted that uncertainty became a constant presence in daily patients’ and HCPs’ lives [30]. In such a situation, women may have felt without a guide (unclear guidance) during the pathway of pregnancy and childbirth. When it came to the media, women in our study reported feeling overwhelmed by the constant stream of conflicting information (infodemic) about COVID-19. Our results align with the findings of Ruiu (2020), who noted that a lack of coordination between political and scientific levels, and between institutional claim-makers and the media, contributed to a sense of mismanagement during the early phases of the COVID-19 outbreak in Italy [31]. In terms of peers and family, as mentioned above, women expressed concern about the safety of social interactions and the potential risk of COVID-19 transmission. These feelings could be affected by the conflicting information during the first phase of the pandemic given by the media. The themes of uncertainty and confusion, linked to the misinformation and inaccuracy themes, were prominent in the experiences of the pregnant and postpartum women in our study, affecting their interactions with HCPs, media, and peers and family. This underscores the need for clear, consistent, and empathetic communication during times of crisis, as well as the importance of providing psychological support.

Regarding women’s relationship with HCPs, competence and reassurance (expressed in the themes of emotional support and reassurance and professionalism and competence) were strongly linked to each other. Pregnant and postpartum women emphasized the importance of skilled and knowledgeable HCPs who are able to give clear and comforting information about COVID-19. These themes could be linked to trust or mistrust towards HCPs and the healthcare system in general. As mentioned above, mistrust also involves the media due to sensationalism and alarmism, which lead to a climate of uncertainty (uncertainty and confusion). Regarding peers and family, sharing information through remote communication technologies helped mothers cope with the situation, which rapidly changed. In this sense, social networks helped women to alleviate stress.

The theme of hope and optimism also emerged as a significant aspect of the emotional landscape in our survey. Regarding communication with HCPs, hope and optimism were often associated with clear and empathetic communication. Dimino et al. (2020) highlighted the importance of the HCPs’ positive psychological state (the so-called PsyCap) characterized by hope, efficacy, resilience, and optimism to guarantee high-quality, evidence-based patient care during the COVID-19 pandemic [32]. They also emphasized the role of nurse leaders in fostering PsyCap in their staff. Finally, sharing experiences, advice, and emotional support with peers and family helped women navigate their emotional responses to the pandemic.

Disappointment and sadness emerged as themes in the open-ended questions section. Women suffered from not being able to experience pregnancy and breastfeeding as they imagined; HCPs should take into account such feelings due to the impact of a mismatch between expectations and experiences of pregnancy on mothers’ psychological well-being [33]. These findings confirm the role of the pandemic in worsening mothers’ mental health.

Loneliness and isolation emerged as significant themes in the experiences of pregnant and postpartum women during the COVID-19 pandemic. These feelings were reported in relation to their interactions with healthcare providers (HCPs), media, and peers and family. In the context of communication with HCPs, women reported feelings of isolation due to the necessary safety measures, such as social distancing and limited face-to-face consultations. This isolation was exacerbated by the lack of physical support systems, such as the presence of a partner or family member during prenatal visits or childbirth. These results are in line with those of Xiang et al. (2020), who noted that patients in quarantine might experience boredom, loneliness, and anger [34]. The media, while providing necessary information, may have contributed to feelings of loneliness and isolation. The constant flow of news about the pandemic, often focusing on the number of cases and deaths, could be linked to a sense of being alone. Moreover, social isolation could be the catalyst for mental health disorders, even in people without a history of psychiatric diseases [35]. These findings underlined the pivotal role of adequate psychological support for mothers during health crises. Finally, for some women, communication with peers and family could be a source of loneliness and isolation if the adaptation to the new normal communication strategies was not achieved.

Finally, the theme of coping strategies is a crucial aspect of the emotional response to the COVID-19 pandemic. Women in our study reported some strategies to manage the emotional toll of the pandemic, as reflected in their interactions with HCPs, media, and peers and family. Women reported using strategies such as seeking support from their HCPs, limiting exposure to distressing news, seeking out positive news stories, and using social media to connect with others. In their communication with peers and family, women reported the adoption of new technologies as a strategy to maintain social connections and to share information with peers and family, which was a helpful strategy to cope with the rapidly changing situation. This is consistent with the findings of Brooks et al. (2020), who noted the importance of maintaining social connections and engaging in self-care activities during periods of quarantine [36]. Moreover, hope and optimism towards aspects such as a healthy baby or a supportive partner seemed to be linked to a more optimistic outlook on the situation (positive mindset). Dispositional optimism is a significant source of coping [37] and, in this sense, further research should deepen the strategies to improve such a characteristic.

Present findings provide a deeper understanding of the complex emotional experiences of pregnant women during the acute phase of the COVID-19 pandemic. The themes of trust, joy, sadness, and fear offer valuable insights into the emotional landscape of these women, highlighting the significant impact of external factors on their emotional well-being. The role of HCPs, media, and peers in shaping these emotions underscores the importance of effective communication and support in enhancing the emotional well-being of pregnant women.

### 4.3. Strengths and Limitations

This study used natural language processing and machine learning methods to classify emotions, identify themes, and extract textual citations from a large national survey database. This approach has some advantages over human coding, such as speed, scalability, and consistency. AI-based coding can process large amounts of data in a relatively short time, handle complex and diverse tasks, and avoid human errors and biases [38].

However, AI-based coding also has some limitations compared to human coding, such as accuracy, interpretability, and creativity. The GPT-3.5 zero-shot learning AI-based coding used here may not yield results as accurate as a fine-tuned model or a model specifically designed for the task. It may also be difficult to explain how the AI model arrived at its results or to verify its validity. Moreover, AI-based coding lacks the human ability to generate novel and original insights from the data or to capture subtle nuances and contextual factors that influence human emotions and behaviors [39,40].

All these limitations notwithstanding, AI-automated classification of pre-COVID and post-COVID emotions in the full sample of the COVID-ASSESS survey, reported in this manuscript, is very consistent with the human-performed preliminary evaluation on the first 200 women answering the survey in April 2022 [15], suggesting satisfactory agreement between the two techniques.

## 5. Conclusions

The results of our research offer a comprehensive exploration of the emotional experiences and coping mechanisms of pregnant and postpartum women in Italy during the initial months of the COVID-19 pandemic. Our findings underscore the significant shift in the emotional landscape induced by the pandemic. Fear and anxiety emerged as dominant emotions, driven by concerns about personal health, the wellbeing of their unborn or newborn child, and the uncertainty surrounding the ongoing global crisis. This highlights the profound psychological impact of the pandemic on this specific group of people, emphasizing the need for targeted mental health support.

Crucially, our study also underscores the importance of communication during such crises. The pandemic exacerbated fear and anxiety, with misinformation and fear-mongering sometimes present in media and social networks. On the other hand, we also found that positive, supportive communication from healthcare professionals, family, and peers played a crucial role in promoting women’s psychological wellbeing.

Reflecting on these findings, it is clear that the COVID-19 pandemic had a profound impact on the emotional experiences of pregnant and postpartum women. As we move forward from this global crisis, it remains crucial to prioritize the mental health of women and provide them with the necessary resources and support. Our research contributes to the discourse on mental health during significant public health crises, particularly during pregnancy and postpartum. We hope that it will inform the development of effective strategies to protect and support such important members of the population in future challenging circumstances.

## Figures and Tables

**Table 1:** General characteristics of the sample.

	Group							
	Pregnant		Postpartum		Total		χ2	*p*
	No.	%	No.	%	No.	%		
**Age classes**								
18–25	15	1.3%	8	1.3%	23	1.3%	3.075	0.380
25–30	157	13.8%	74	11.6%	231	13.0%		
30–35	436	38.4%	268	42.0%	704	39.7%		
>35	528	46.5%	288	45.1%	816	46.0%		
**Level of education**								
Lower secondary education	38	3.4%	25	4.0%	63	3.5%	5.884	0.436
Upper secondary education	291	25.6%	180	28.2%	471	26.6%		
Post-secondary non-tertiary education	289	25.4%	148	23.2%	437	24.6%		
First stage of tertiary education	322	28.3%	194	30.4%	516	29.1%		
Second stage of tertiary education	196	17.3%	91	14.3%	287	16.2%		
**Trimester** (pregnant only)								
First	113	9.9%						
Second	479	42.2%						
Third	544	47.9%						
**Baby age** (months, postpartum only)								
<1.4			243	38.1%				
1.5–3.9			194	30.4%				
>4			201	31.5%				
**Number of previous pregnancies**								
Multigravidae	745	65.6%	394	61.8%	1139	64.2%	2.602	0.107
First pregnancy	391	34.4%	244	38.2%	635	35.8%		
**Personal history**								
Psychopathological history	499	43.9%	291	45.6%	790	44.5%	0.470	0.493
**Other children (n)**								
0	565	49.7%	341	53.4%	906	51.1%	3.493	0.322
1	500	44.0%	252	39.5%	752	42.4%		
2	56	4.9%	36	5.6%	92	5.2%		
>2	15	1.3%	9	1.4%	24	1.4%		
**Previous pregnancy loss**								
No previous loss	692	60.9%	407	63.8%	1099	62.0%	1.435	0.231
Previous loss	444	39.1%	231	36.2%	675	38.0%		

## Data Availability

The data presented in this study are openly available in Mendeley Data at 10.17632/cn38pbwn7r.1.

## Supplementary Materials

The following supporting information can be downloaded at https://www.mdpi.com/article/10.3390/jcm12196140/s1, Table S1: Word count and frequency of emotions before and during the pandemic.

## References

[1] Remuzzi A., Remuzzi G. (2020). COVID-19 and Italy: What next?. Lancet.

[2] Rossi R., Socci V., Talevi D., Mensi S., Niolu C., Pacitti F., Di Marco A., Rossi A., Siracusano A., Di Lorenzo G. (2020). COVID-19 Pandemic and Lockdown Measures Impact on Mental Health Among the General Population in Italy. Front Psychiatry.

[3] Carpiniello B., Vita A. (2022). Impact of COVID-19 on the Italian Mental Health System: A Narrative Review. Schizophr. Bull. Open.

[4] Lu X., Lin Z. (2021). COVID-19, Economic Impact, Mental Health, and Coping Behaviors: A Conceptual Framework and Future Research Directions. Front Psychol..

[5] Loughnan S.A., Gautam R., Silverio S.A., Boyle F.M., Cassidy J., Ellwood D., Homer C., Horey D., Leisher S.H., de Montigny F. (2022). Multicountry study protocol of COCOON: COntinuing Care in COVID-19 Outbreak global survey of New, expectant, and bereaved parent experiences. BMJ Open.

[6] Villar J., Ariff S., Gunier R.B., Thiruvengadam R., Rauch S., Kholin A., Roggero P., Prefumo F., Do Vale M.S., Cardona-Perez J.A. (2021). Maternal and neonatal morbidity and mortality among pregnant women with and without COVID-19 infection: The INTERCOVID multinational cohort study. JAMA Pediatr..

[7] Filippetti M.L., Clarke A.D.F., Rigato S. (2022). The mental health crisis of expectant women in the UK: Effects of the COVID-19 pandemic on prenatal mental health, antenatal attachment and social support. BMC Pregnancy Childbirth.

[8] Ahmad M., Vismara L. (2021). The Psychological Impact of COVID-19 Pandemic on Women’s Mental Health during Pregnancy: A Rapid Evidence Review. Int. J. Environ. Res. Public Health.

[9] Salehi L., Rahimzadeh M., Molaei E., Zaheri H., Saeieh S.E. (2020). The relationship among fear and anxiety of COVID-19, pregnancy experience, and mental health disorder in pregnant women: A structural equation model. Brain. Behav..

[10] White T., Matthey S., Boyd K., Barnett B. (2006). Postnatal depression and post-traumatic stress after childbirth: Prevalence, course and co-occurrence. J. Reprod Infant Psychol..

[11] Czarnocka J., Slade P. (2000). Prevalence and predictors of post-traumatic stress symptoms following childbirth. Brit. J. Clin. Psychol..

[12] Mental Health and Substance Use. https://www.who.int/teams/mental-health-and-substance-use/promotion-prevention/maternal-mental-health.

[13] Khoury J.E., Atkinson L., Bennett T., Jack S.M., Gonzalez A. (2021). Coping strategies mediate the associations between COVID-19 experiences and mental health outcomes in pregnancy. Arch. Womens Ment Health.

[14] Khoury J.E., Atkinson L., Bennett T., Jack S.M., Gonzalez A. (2021). COVID-19 and mental health during pregnancy: The importance of cognitive appraisal and social support. J. Affect Disord..

[15] Ravaldi C., Wilson A., Ricca V., Homer C., Vannacci A. (2020). Pregnant women voice their concerns and birth expectations during the COVID-19 pandemic in Italy. Women Birth.

[16] Ravaldi C., Ricca V., Wilson A., Homer C., Vannacci A. (2020). Previous psychopathology predicted severe COVID-19 concern, anxiety, and PTSD symptoms in pregnant women during ‘lockdown’ in Italy. Arch. Womens Ment. Health.

[17] Ravaldi C., Mosconi L., Wilson A.N., Amir L.H., Bonaiuti R., Ricca V., Vannacci A. (2022). Exclusive breastfeeding and women’s psychological well-being during the first wave of COVID-19 pandemic in Italy. Front. Public Health.

[18] Ravaldi C., Vannacci A. (2020). The COVID-ASSESS dataset—COVID19 related anxiety and stress in prEgnancy, poSt-partum and breaStfeeding during lockdown in Italy. Data Brief..

[19] Ravaldi C., Vannacci A. (2020). COVID-ASSESS Italy—COVID19 related Anxiety and StreSs in prEgnancy, poSt-partum and breaStfeeding. Mendeley.

[20] Introducing ChatGPT. https://openai.com/blog/chatgpt.

[21] Wu T., He S., Liu J., Sun S., Liu K., Han Q.L., Tang Y. (2023). A Brief Overview of ChatGPT: The History, Status Quo and Potential Future Development. IEEE/CAA Autom. Sin..

[22] GPT-4. https://openai.com/gpt-4.

[23] Di Guardo F., Di Grazia F.M., Di Gregorio L.M., Zambrotta E., Carrara G., Gulino F.A., Tuscano A., Palumbo M. (2021). Poor maternal-neonatal outcomes in pregnant patients with confirmed SARS-Cov-2 infection: Analysis of 145 cases. Arch. Gynecol. Obs..

[24] Bell A.F., Andersson E. (2016). The birth experience and women’s postnatal depression: A systematic review. Midwifery.

[25] Henriksen L., Grimsrud E., Schei B., Lukasse M. (2017). Factors related to a negative birth experience—A mixed methods study. Midwifery.

[26] Smorti M., Gemignani A., Bonassi L., Mauri G., Carducci A., Ionio C. (2022). The impact of Covid-19 restrictions on depressive symptoms in low-risk and high-risk pregnant women: A cross-sectional study before and during pandemic. BMC Pregnancy Childb.

[27] Azoulay E., Pochard F., Reignier J., Argaud L., Bruneel F., Courbon P., Cariou A., Klouche K., Labbé V., Barbier F. (2021). Symptoms of Mental Health Disorders in Critical Care Physicians Facing the Second COVID-19 Wave: A Cross-Sectional Study. Chest.

[28] Infodemic. https://www.who.int/health-topics/infodemic.

[29] Sasaki N., Kuroda R., Tsuno K., Kawakami N. (2020). Exposure to media and fear and worry about COVID-19. Psychiatry Clin. Neurosci..

[30] Koffman J., Gross J., Etkind S.N., Selman L. (2020). Uncertainty and COVID-19: How are we to respond?. J. R. Soc. Med..

[31] Ruiu M.L. (2020). Mismanagement of COVID-19: Lessons learned from Italy. J. Risk Res..

[32] Dimino K., Horan K.M., Stephenson C. (2020). Leading Our Frontline HEROES Through Times of Crisis with a Sense of Hope, Efficacy, Resilience, and Optimism. Nurse Lead.

[33] Webb R., Ayers S., Bogaerts A., Jeličić L., Pawlicka P., Van Haeken S., Uddin N., Xuereb R.B., Kolesnikova N. (2021). When birth is not as expected: A systematic review of the impact of a mismatch between expectations and experiences. BMC Pregnancy Childb..

[34] Xiang Y.T., Yang Y., Li W., Zhang L., Zhang Q., Cheung T., Ng C.H. (2020). Timely mental health care for the 2019 novel coronavirus outbreak is urgently needed. Lancet Psychiatry.

[35] Usher K., Bhullar N., Jackson D. (2020). Life in the pandemic: Social isolation and mental health. J. Clin. Nurs..

[36] Brooks S.K., Webster R.K., Smith L.E., Woodland L., Wessely S., Greenberg N., Rubin G.J. (2020). The psychological impact of quarantine and how to reduce it: Rapid review of the evidence. Lancet.

[37] Niewiadomska I., Bień A., Rzońca E., Jurek K. (2022). The Mediating Role of Dispositional Optimism in the Relationship between Health Locus of Control and Self-Efficacy in Pregnant Women at Risk of Preterm Delivery. Int. J. Environ. Res. Public Health.

[38] Jiang F., Jiang Y., Zhi H., Dong Y., Li H., Ma S., Wang Y., Dong Q., Shen H., Wang Y. (2017). Artificial intelligence in healthcare: Past, present and future. Stroke Vasc. Neurol..

[39] Alm C.O., Hedges A. (2021). Visualizing NLP in Undergraduate Students’ Learning about Natural Language. AAAI.

[40] Deng J., Lin Y. (2023). The Benefits and Challenges of ChatGPT: An Overview. FCIS.

