# Supplementary material for: The Emotional Landscape of Pregnancy and Postpartum during the COVID-19 Pandemic in Italy: A Mixed-Method Analysis Using Artificial Intelligence
Source: J Clin Med. 2023 Sep 23;12(19):6140. doi: 10.3390/jcm12196140 (PMC10573687; doi:10.3390/jcm12196140)
Supplement: Supplementary file 1 [file jcm-12-06140-s001.zip › jcm-2617158-supplementary.pdf]

Table S1. Word count and frequency of emotions before and during the pandemic.

## Pre – Covid

Total: 3850 words

| Weight | Word | Italian words and sinonyms |
|---|---|---|
| **276** | Happiness | felicità, felice |
| **196** | Joy | gioia |
| **192** | Exciting | emozionante, eccitazione, eccitato |
| **149** | Emotion | emozione, emozioni |
| **138** | Shared | condiviso |
| **104** | Sharing | condivisione, condividere |
| **102** | Family | famiglia |
| **101** | Calm | sereno, calmo |
| **96** | Painful | doloroso |
| **89** | Tranquility | tranquillo, tranquilla, tranquillità |
| **84** | Husband | marito |
| **83** | Pain | dolore |
| **69** | Love | amore |
| **67** | Serenity | serenità |
| **54** | Fear | paura |
| **37** | Joyful | gioioso |
| **37** | Safety | sicurezza |
| **24** | Support | sostegno, supporto |
| **21** | Anxiety | ansia |
| **20** | Intense | intenso |
| **18** | Hope | speranza |
| **17** | Exhausting | faticoso |
| **16** | Festivity | festa |
| **16** | Assisted | assistito |
| **15** | Relatives | parenti |
| **12** | Life | vita |
| **12** | Union | unione |
| **12** | Water | acqua |
| **11** | Unforgettable | indimenticabile |
| **11** | Welcome | accoglienza |
| **10** | Friends | amici |
| **10** | Relieving | liberatorio |

## Post – Covid

Total: 3872 words

| Weight | Word | Italian words and sinonyms |
|---|---|---|
| **319** | Fear | paura, pauroso, terrore, spavento, timore |
| **190** | Anxiety | ansia, ansiogeno, ansioso, angoscia, agitato, angosciante, inquietudine, tensione, apprensione |
| **179** | Loneliness | solitudine, isolamento, solitario, sola, isolato, abbandonata, solitaria |
| **34** | Joy | felicità, gioia, felice, gioioso |
| **32** | Excitement | emozionante, coinvolgente, appagante, elettrizzante |
| **25** | Hope | speranza, speranzosa, ottimismo |
| **25** | Uncertainty | imprevedibilità, incertezza, incerto |
| **20** | Distance | lontananza, distanza |
| **19** | Concern | preoccupante, preoccupata, preoccupazione |
| **19** | Detachment | distacco, separata, separazione |
| **16** | Family | famiglia, marito, padre, figlio, bambino, coniuge, figlia, genitori, partner, madre, nonna, materna |
| **12** | Contagion | contagio |
| **10** | Danger | pericolo, pericoloso |
| **10** | Risk | rischio |
| **8** | Coldness | freddezza, freddo |
| **4** | Hurried | frettoloso, affrettato |
| **4** | Restrictions | restrittivo, restrizioni |
| **3** | Disappointment | delusione, delusa |

Full list of words is published as raw data in Ravaldi & Vannacci (2020) *COVID-ASSESS Italy—COVID19 related Anxiety and StreSs in prEgnancy, poSt-partum and breaStfeeding* [Data set]. Mendeley. https://doi.org/10.17632/CN38PBWN7R.1
